# Tailoring Therapy to Bronchopulmonary Dysplasia Phenotype: A Ten-Year Experience in Precision Medicine

**DOI:** 10.3390/children13020275

**Published:** 2026-02-17

**Authors:** Arvind Sehgal, Samuel Menahem

**Affiliations:** 1 Monash Newborn, Monash Children’s Hospital, Melbourne, VIC 3168, Australia; 2 Department of Paediatrics, Monash University, Melbourne, VIC 3168, Australia; 3 Emeritus Head, Paediatric and Fetal Cardiac Units, Monash Medical Centre, Monash Health, Melbourne, VIC 3168, Australia; samuel.menahem@monash.edu

**Keywords:** ACE inhibition, chronic lung disease, captopril, pulmonary hypertension

## Abstract

**Highlights:**

**What are the main findings?**

- Conventionally, pulmonary artery hypertension is known in infants with severe bronchopulmonary dysplasia.
- Diagnostic and therapeutic emphasis is on right-sided haemodynamic alterations.

**What are the implications of the main findings?**

- Systemic hypertension–systemic artery stiffness–left heart dysfunction complicating a subset of such infants are not well appreciated.
- Systemic afterload reduction with ACE inhibitors such as captopril is physiologically more suited to this phenotype.
- Pulmonary vasodilators such as inhaled nitric oxide may cause deterioration.

**Abstract:**

Aims: To assess the impact of systemic afterload reduction on cardiorespiratory health in infants with a severe bronchopulmonary dysplasia (BPD)–systemic hypertension–left heart dysfunction (LHD) phenotype. Methods: Ten-year data were prospectively collected and analysed. The cohort included extremely preterm infants with severe BPD–systemic hypertension–LHD pathophysiology, treated with systemic afterload reduction with captopril. Main outcome measures included improvement in oxygenation and echocardiographic measures of LHD. Results: Twenty-six infants with gestation of 26.5 ± 2 weeks and median (interquartile range) birthweight of 900 (582, 1083) g were administered captopril at the corrected gestation median (range) of 40 weeks (37–67). On reassessment after five weeks, oxygen requirements (43 ± 16% to 26 ± 7%, *p* = 0.0001) and paired pCO_2_ decreased (64 ± 9 to 53 ± 9 mm Hg, *p* = 0.0001). Significant improvements were seen in cardiac indices (diastolic: trans-mitral E/A ratio [1 ± 0.07 to 0.94 ± 0.07, *p* = 0.0004] and iso-volumic relaxation time [ms] [65 ± 3 to 56 ± 4, *p* < 0.0001], and systolic: mean velocity of circumferential fibre shortening [circ/s] [1.6 ± 0.2 to 1.9 ± 0.2, *p* < 0.0001] and left ventricular output [mL/kg/min] [177 ± 34 to 230 ± 54, *p* = 0.0002]). This coincided with improved aortic pulsatility (40 ± 13 to 50 ± 11 µm, *p* = 0.005). Conclusions: Systemic hypertension–LHD pathology amongst infants with severe BPD may be under-recognised. Systemic afterload reduction is physiologically suited in a subset of infants with severe BPD.

## 1. Introduction

Infants delivered at less than 28 weeks gestational age (GA) have a consistently high incidence of severe bronchopulmonary dysplasia (BPD) (defined as oxygen requirement of ≥30% and/or positive pressure respiratory support at 36-week postmenstrual age [PMA]), as reported by the National Institute of Child Health and Human Development Neonatal Research Network over the last two decades [1,2]. In terms of our understanding of the involved cardiovascular issues, the right heart has been the focus of diagnosis and therapy. Systemic hypertension and/or systemic arteries stiffness (increased systemic afterload) together with left heart dysfunction (LHD), sequentially or in unison, may be mechanistically relevant and therapeutically germane in BPD pathophysiology. Left ventricular (LV) maturational issues put premature infants at high risk for ongoing LHD [3,4]. In an earlier study, extremely preterm infants with severe BPD (born at <28 weeks and evaluated at 36 weeks PMA) were found to have LHD (both diastolic and systolic), compared to equally premature infants with no BPD or term-born asymptomatic infants [3]. Recent data also found systemic hypertension in infants with severe BPD, which was associated with an increased duration of respiratory support requirements [5,6]. Amongst infants with severe BPD, those with systemic hypertension also showed evidence of LHD [7]. Systemic hypertension and systemic arteries stiffness as a cause of elevated afterload may lead to an oedemagenic response of the lungs via a backpressure effect, ‘the post-capillary pathophysiology’ [3,4,6,7,8,9] (Supplementary Online Figure S1). However, how systemic hypertension, the impairment in LV function, and respiratory morbidity might suggest possible therapeutic options is poorly understood.

Lack of appreciation of this phenotype may lead to unanticipated adverse effects from physiologically inappropriate treatment. For instance, pulmonary vasodilators such as inhaled nitric oxide (iNO) may increase the likelihood of pulmonary oedema as well as its severity. Preliminary data showed potential benefits from angiotensin-converting enzyme (ACE) inhibition in a subset of infants with severe BPD [10,11,12]. A better appreciation of the underlying principles could make systemic afterload reduction with ACE inhibition a more appropriate physiological treatment strategy.

The objective of this study was to evaluate the impact of captopril on cardiorespiratory health and LHD in premature infants with a severe BPD–systemic hypertension–LHD phenotype.

## 2. Methods

This study was conducted at a 64-bed quaternary neonatal intensive care unit, caring for infants ≥22 weeks GA in addition to infants requiring surgery. Approximately 100 infants weighing <1000 g at birth are admitted annually. Over the last 10 years, this included approximately 13 infants/year with severe BPD. Since 2015, cardiorespiratory and echocardiography data on infants with severe BPD and associated systemic hypertension treated with captopril have been systematically collected. These data, prospectively collected between July 2015 and July 2025 (ten years), have since been analysed following ethics approval (Monash Health HREC 478902). Echocardiography screening for chronic PH in preterm infants with severe BPD is routinely performed at 36 weeks PMA (or earlier if clinically indicated).

For the purpose of this study, a single investigator (AS) using the Vivid E95 Advantage cardiovascular ultrasound system (GE Medical Systems, Milwaukee, WI, USA) performed all echocardiograms before and five weeks after captopril administration. Analyses were performed using a dedicated EchoPAC^TM^ (GE Vingmed Ultrasound, Horten, Norway) workstation according to published methodology [13,14]. Three to five consecutive cardiac cycles were evaluated and averaged for each measurement. Echocardiographic indices, views and cursor position for measurements have been previously summarized [3,8,13,14,15,16]. These included measures of systolic function (fractional shortening, mean velocity of circumferential fibre shortening, cardiac output, LV tissue Doppler-derived myocardial performance index, tricuspid annular plane systolic excursion and pulmonary vascular resistance [PVR] index (1/[TPV/RVET]). Measures of diastolic function included trans-mitral E/A ratio, iso-volumic relaxation time, mitral valve stroke volume and pulmonary vein velocity time integral. Aortic properties evaluated included intima-media thickness and pulsatility (difference between systolic and diastolic dimension) at the straight, unbranched, longitudinal section of the abdominal aorta as reported previously [17]. The arterial wall stiffness index (β index) was calculated using the following formula: ln (systolic BP/diastolic BP)/[(AAOs-AAOd)/AAOd], where AAOs and AAOd are internal aortic dimensions in systole and diastole, respectively. Blood pressure (BP) measurements were performed by the oscillometric method, concurrent at each echocardiography assessment.

Infants for whom captopril was administered for the management of heart failure arising from congenital heart disease and/or systemic hypertension not accompanied by BPD were excluded. Congenital heart disease including pulmonary vein stenosis was excluded by echocardiograms performed by the paediatric cardiology service.

### 2.1. Unit Protocol for Captopril Use for Systemic Hypertension

Our standard unit practice was undertaken, namely, to administer captopril for systemic hypertension in consultation with the renal and cardiac physicians, and to have informed discussions with parents when the systolic BP is consistently >95th centile for PMA [18]. Pre-captopril investigations including a complete blood count, serum urea, creatinine and electrolytes, and a renal ultrasound were performed, and were reported within normal limits. To avoid significant first dose hypotension, a reduced first dose was administered before titrating to the target dose [19,20,21,22]. The initial dose of captopril was 0.01 mg/kg/dose administered orally every eight hours and increased to 0.1 mg/kg/dose by the end of the first week. Subsequent increases were at 5–7-day intervals to reach 0.5 mg/kg/dose. The time to the peak anti-hypertensive effect is 60–90 min after oral ingestion; however, the time to optimal anti-hypertensive effect following regular administration at a therapeutic dose is many weeks after commencement [20,21,22,23]. While infants were under continuous cardiorespiratory monitoring, we a priori planned to repeat clinical and echocardiographic assessments after an interval of five weeks. This timescale would allow for reaching the target maximum dose and expected accumulation of captopril metabolites. While data concerning hypertension during infancy is limited, pharmacokinetic parameters for captopril in infants with congestive heart failure have been reported within the range seen in adults with heart failure [21]. Renal functions are monitored weekly until discharge.

Clinical data collected included neonatal demographics (GA, birthweight, sex, and antenatal steroids), antecedent therapies such as surfactant, iNO, treatment for patent ductus arteriosus (PDA), postnatal steroid administration (DART regimen [24]), sildenafil, and the clinical response to captopril. Respiratory status data (ventilation, fraction of inspired oxygen [FiO_2_] and pCO_2_) were collected prior to and then after five weeks of captopril. For infants on low-flow oxygen, effective FiO_2_ was recorded [25]. Clinical data accessed were what was submitted to the Australian and New Zealand Neonatal Network registry. Captopril dosage was obtained from Monash Health Pharmacy Informatics. Echocardiography data was stored in EchoPAC^TM^ as coded but, if required, re-identifiable information. The outcomes evaluated included changes in clinical indices of cardiorespiratory health and echocardiography indices of cardiovascular function.

### 2.2. Statistics

Analyses were performed using the Stata/BE 17 software (StataCorp, College Station, TX, USA). Descriptive statistics were used to analyse demographics and clinical data. The Shapiro–Wilk test was used to evaluate continuous variables for normality. Mean ± standard deviation and median (interquartile) range were calculated for data with normal and non-normal distributions, respectively. Categorical variables were presented as frequencies (%). Comparative evaluation of pre- and post-captopril echocardiographic variables was performed using a paired *t*-test. Statistical significance was regarded as two tailed *p* < 0.05. No sample size calculation was performed as there were insufficient earlier data regarding the impact of captopril on LV function in the setting of BPD and systemic hypertension. A sample size of convenience was used for this hypothesis-generating study, aiming to analyse data over a ten-year period.

## 3. Results

During the study period, 51 infants were administered captopril. Amongst them, 21 infants with congenital heart disease-associated cardiac failure, three infants with non-BPD-accompanying hypertension, and one infant where paired echocardiographic data was not available, were excluded. Further analysis involved the remaining 26 infants. The GA and birthweight of the cohort were 26.5 ± 2 weeks and median (interquartile range) 900 (582, 1083) g, respectively. Table 1 summarizes these demographics and the use of other medications such as sildenafil, the timing and GA when captopril was administered, and respiratory status at the time of discharge. Six infants (23%) had previously been administered iNO in the first four weeks of their life; none was on it at the time of captopril administration. All infants had been managed with conventional and/or high frequency ventilation in the lead up to captopril administration. Twelve (46%) infants had been previously prescribed postnatal dexamethasone though none was on it at the time of captopril administration. Twelve infants had been administered paracetamol for medical closure of PDA; none required surgical/device closure. A small duct (<1 mm) was present in three infants at the time of captopril administration.

Clinical outcomes: There were 24/26 (92%) survivors; the ventilation modes at the start of captopril were high-frequency ventilation (one), mechanical intermittent mandatory ventilation (five) and continuous positive airway pressure (eighteen) infants. The two non-survivors were on high frequency ventilation. Ventilation modes for the survivors at the end of five weeks of captopril were intermittent mandatory ventilation (one), continuous positive airway pressure (eight), low-flow oxygen (six) and self-ventilating in room air (nine). The FiO_2_ decreased from 0.43 ± 0.16 to 0.26 ± 0.07, *p* = 0.0001, while the pCO_2_ on paired blood gas fell from 64 ± 9 to 53 ± 9 mm Hg, *p* = 0.0001. The systolic BP also fell from >95th centile (>100 mm Hg in all cases) to between the 50th and 95th centile, closer to the 50th centile at the repeat assessments. The mean (range) systolic BP at five weeks for the cohort was 72 (62–89) mm Hg. Improvements in respiratory status (FiO_2_ and ventilator pressure requirements) were observed by the end of the second week, and preceded normalisation of BP. We evaluated the correlation between systolic BP at five weeks of therapy with LV stroke volume and output. The correlation was r = −0.38, *p* = 0.005 for stroke volume and r= −0.4, *p* = 0.04 for output, signifying that a reduction in systolic BP was associated with an improved LV performance. All surviving infants (except one) were discharged on captopril, which was weaned over the subsequent 3–6 months during follow up. Two infants died during the stay due to hypoxia from severe lung disease; both had intrauterine growth restriction (birthweight <10th centile for GA). No infant developed cough or electrolyte abnormalities after captopril administration.

**Figure 1:**
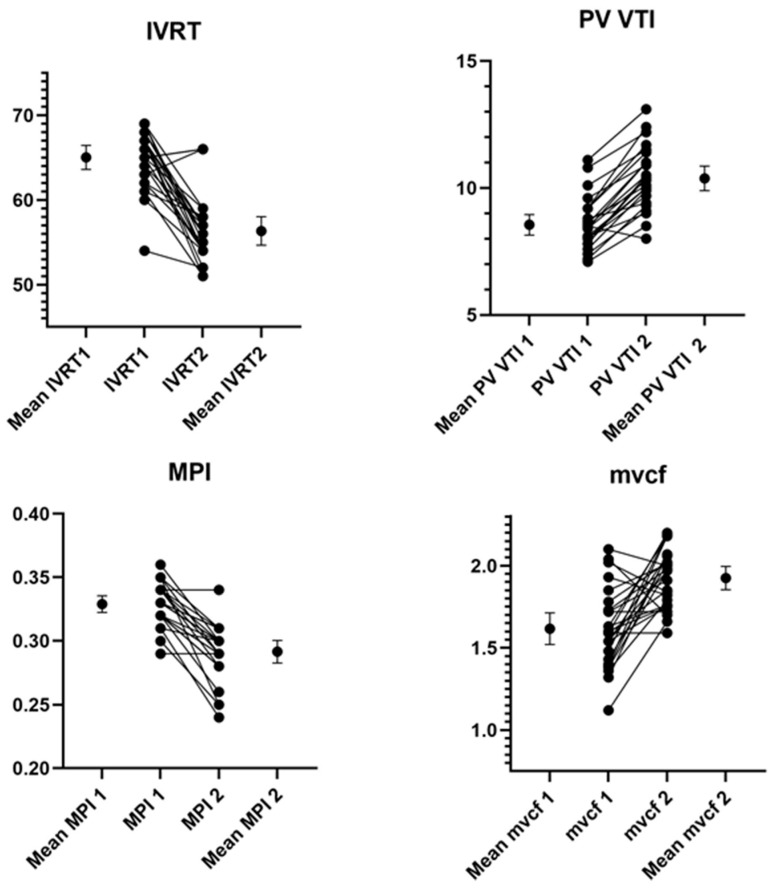
Evolution of individual patient echocardiography indices noting improvements in diastolic and systolic function. IVRT—Iso-volumic.

Echocardiographic outcomes: Pathological tricuspid regurgitation (≥2.8 m/s) was not seen in the cohort. The foramen *ovale* was closed in 19 infants and restrictive in the rest. Table 2 summarizes the echocardiographic indices before and five weeks after captopril administration; improved relaxation and contractility were noted. Improved LV diastolic function and easing of end-diastolic left atrial pressure were associated with improved trans-mitral and pulmonary venous flow. There was coincident reduction in the PVR index and improved right ventricular output. Improved pulsatility in the abdominal aorta (beat to beat variation between systole and diastole) was noted. The stiffness index noted a statistically non-significant lowering (3.2 ± 0.7 vs. 2.8 ± 0.7. *p* = 0.09). Supplementary Online Figure S2 depicts baseline echocardiographic information and its evolution with captopril therapy in one representative infant. Figure 1 depicts the change in echocardiographic parameters in individual infants.

## 4. Discussion

Our study noted improvements in cardiorespiratory health and echocardiographic indices in infants with a severe BPD–systemic hypertension–LHD phenotype following systemic afterload reduction. Functional classification of PH in childhood now allows for identifying a subset where LHD is a contributor [26,27]. Complex BPD pathogenesis requires enhanced diagnostic precision, enabling judicious therapeutic choices appropriate for the underlying phenotype. Supplementary Online Table S1 describes various acute and chronic PH phenotypes, including BPD–systemic hypertension–LHD phenotype (‘post-capillary pathophysiology’).

### 4.1. Systemic Hypertension–Arterial Stiffness–LHD: A Less Recognized BPD Phenotype

Recognition of LHD-BPD phenotype amongst infants with severe BPD has gained traction over the last decade. However, most studies have been diagnostic in nature; therapeutic evidence remains in the form of small case series [3,4,9,11,12,28]. LHD is characterized by increased LV stiffness and abnormal relaxation, which leads to elevated LV filling pressures and abnormal diastolic filling, leading to pulmonary venous congestion contributing to a continued need for respiratory support. In a cross-sectional study, cardiac indices reflecting systemic afterload and pulmonary venous backpressure were systematically evaluated in premature infants with severe BPD [3]. In evaluations done at 36 weeks PMA, significantly impaired LV diastolic function was noted in the BPD group, compared to infants with no BPD. Mourani and colleagues reported two infants with severe BPD where LV diastolic dysfunction contributed to clinical symptomatology (dependence on respiratory support and oxygen requirements) [4]. An earlier catheterization study in preterm infants with severe BPD noted LHD contributed to PH in 58% of the infants [9]. While catheterization is the gold standard, it is not readily available and may not be tolerated by critically ill infants. Through bedside echocardiography, we collected cardiac and arterial information indicating significant contribution from LHD.

The infants in our study had systolic BP >95th centile, and it acted as the clinical trigger to explore BPD-LHD pathophysiology. A high incidence of systemic hypertension has been previously reported in infants with severe BPD [5,6]. Systemic hypertension in this cohort has been associated with respiratory morbidities; putatively linked to backpressure changes to the lungs (arising from the raised pulmonary venous pressure—‘the cardiac–lung disease’). As part of our routine echocardiographic protocol, we evaluated aortic dynamic properties as contributors to elevated systemic afterload. Aortic pulsatility improved in the cohort as noted in the post-captopril evaluations. Increased systemic arteries stiffness and thickness have been previously noted in cohorts with severe BPD in comparison to equally preterm infants with no BPD [8]. Studies in adults have evaluated arterial thickness longitudinally where it served as an end-point in clinical trials, with a strong predictive value for cardiovascular disease events beyond that of the classical risk factors such as increased pulse pressure [29,30]. Dynamic vascular studies and their clinical correlates represent an emerging field of research in paediatric medicine [3,31,32]. Whether it can become an end-point of therapeutic studies amongst neonatal-paediatric cohorts remains to be explored. Alongside increased systemic artery stiffness, elevated levels of circulating catecholamines and reactive oxygen species, with an alteration in arterial wall elastin–collagen ratio (elastin being replaced by collagen which is 100 times stiffer), are factors which elevate the systemic afterload—potentially adversely impacting left heart function.

### 4.2. Therapeutic Constructs Based on BPD Phenotype

Information derived from adult literature is quite informative as similar pathophysiology is found in >60% of patients with LV systolic dysfunction (and >80% in patients with diastolic dysfunction) [33,34]. The incidence of ‘post-capillary’ pathophysiology BPD phenotype is unclear. Of approximately 13 infants/year with severe BPD in our neonatal intensive care unit, the findings of this study spanning ten years suggest that it may well be (26/130) 20%. A recent echocardiographic study over four years found a similar estimate [35]. An earlier study of 13 preterm infants with severe BPD evaluated by cardiac catheterization found that LHD contributed to PH in seven (58%) infants [9]. Given catheterization has greater sensitivity compared with echocardiography, our estimates could be an underestimation. Systematic assessments comparing the two modalities in larger cohorts are recommended.

We noted clinical (respiratory support requirements) and echocardiographic (cardiac and arterial pulsatility) improvements in our cohort. The effects of oral captopril on pulmonary haemodynamics have been studied in adult patients with chronic respiratory failure [36]. Significant decreases in pulmonary capillary wedge pressure and ‘systemic’ arterial resistance with no change in PVR indicated a relatively selective systemic effect [36]. However, long-term ACE inhibition did result in a decrease in PVR [37]. Emerging literature supports its potential use as a therapeutic agent in BPD–systemic hypertension–LHD phenotype [12,28]. Such targeted therapies may offer a novel approach to infants with severe BPD and cardiac–lung disease. Mourani and colleagues demonstrated improved clinical and echocardiographic parameters with ACE inhibition in two infants with severe BPD-LHD unresponsive to diuretics and pulmonary vasodilators [4]. Another case series, which included six extremely preterm infants with severe BPD–systemic hypertension–LHD unresponsive to conventional therapies, noted significant reduction in respiratory support requirements and improved cardiac function and aortic pulsatility with ACE inhibition [12]. In a small single-centre observational study on 11 preterm infants with BPD–systemic hypertension–LHD, enalapril was administered at a postnatal age of 90 ± 20 days, which lead to improved LV diastolic function [11]. Our study confirmed this effect in a larger cohort, over an extended time period (ten years). As a part of refinement of therapeutic strategy over time, we found our use of sildenafil and dexamethasone (postnatal steroids) in these infants decreased prior to settling on the physiologically most appropriate therapy of ACE inhibition. In other words, the therapeutic approach evolved to include the various BPD phenotypes in mind rather than following a regimented approach. Over the last ten years, we have systematically worked to establish a more correct pathophysiology-guided approach, which was upheld in the current study.

### 4.3. Therapeutic Rationale: Addressing the Imbalance Between Vasoconstrictors and Vasodilators

The imbalance between the angiotensin-converting enzyme (ACE, vasoconstrictors-proliferators) and ACE2 pathways (Ang-[1–7], vasodilators, anti-proliferators), plays a role in remodelling the vasculature as ACE2 is a counter-regulatory enzyme of ACE [38,39,40]. ACE plays a critical role in the pathogenesis of neonatal lung diseases as well as inducing programmed hypertension [41,42,43]. In animal models, ACE inhibition mediated arterial wall remodelling by inhibiting smooth muscle hypertrophy and replacement of elastin by collagen [44,45], resulting in reduction of the media-to-lumen ratio [46]. Combination of suppression of the ACE/Ang II and activation of the ACE2/Ang (1–7) pathway leads to attenuation of hypoxic PH in rats [47].

### 4.4. Towards Diagnostic and Therapeutic Precision in BPD

Given that the clinical differentiation of the complex pathophysiology of the various BPD phenotypes may be challenging, we put forward a ‘precision medicine’ framework [48,49,50,51] (Supplementary Online Table S2). At the heart of the framework are the integrated team meetings (done monthly, more frequently if required) at our institution.

Serial evolution of echocardiographic indices of LHD alongside clinical improvements support the concept of diagnostic and therapeutic precision. While a decrease in PVR should be expected in the treatment of pulmonary hypertension (PH) when challenged with iNO/100% oxygen, a recent study noted that 7/20 (35%) infants did not demonstrate such a response (≥20% change in indexed PVR) [52]. In this setting, long-term administration of pulmonary vasodilators may be unsuitable, increasing the need for further respiratory support. For example, adult patients with PH and LHD (in the form of mitral valve disease) are excluded from clinical trials evaluating pulmonary vasodilator therapies [52]. Acute vasodilator testing using a brief iNO/100% oxygen challenge may assist in deciphering best practice. A recent survey of PH specialists noted that 41% of the participants would use it in their decision-making [10].

## 5. Limitations

This is a relatively small single-centre cohort study, which may limit generalizability. Nevertheless, it is the largest case series of ‘post-capillary BPD phenotype’ demonstrating therapeutic benefit. There is an inherent selection bias in the cohort as we stayed focused on the relevant phenotype of severe BPD–systemic hypertension–LHD, rather than studying all infants who were administered captopril. Infants were followed up clinically with no follow-up echocardiograms post-discharge. Captopril was gradually weaned based on BP monitoring, with no relapse in respiratory support requirements. Five infants were on sildenafil at the time of captopril; unfortunately, low numbers do not permit multivariate regression analysis. With a multi-centre design and larger numbers in the cohort, further detailed analysis could be performed. We acknowledge that these infants may have shown improvement over time in the absence of captopril treatment, but in our informed opinion, the combined improvement in clinical and echocardiographic parameters is highly suggestive of a genuine treatment effect of pharmacologic efficacy in addressing the appropriate LHD disease phenotype. Finally, this question is best addressed in the setting of randomized controlled trials.

## 6. Conclusions

Our data suggest that in a subset of infants with severe BPD, the cardiorespiratory trajectory may be potentially modifiable with ACE inhibition. As continued respiratory support in infants with severe BPD may be parenchymal or circulatory in its predominant origin, delineating the phenotype should guide therapeutic options. We recommend that infants with severe BPD be systematically evaluated, across multiple centres which manage this cohort. This would enable a data-registry of larger numbers. Strengths of this study include the identification of a specific BPD phenotype, rigorous echocardiographic evaluation, and a targeted therapeutic strategy resulting in meaningful cardiorespiratory improvement.

## Figures and Tables

**Table 1:** Demographic variables of the study cohort n = 26.

Variable	
Gestational age at birth (weeks)	26.5 ± 2
Birthweight (g) ^$^	900 (582, 1083)
Apgar score at 5 min (median [range]) ^$^	7 (6, 9)
Male sex n (%)	17 (65)
Mode of delivery, Caesarean section n (%)	16 (61)
Intubation at birth n (%)	13 (50)
Antenatal steroids n (%)	21 (81)
Premature rupture of membranes > 7 days	0
Surfactant replacement therapy n (%)	22 (85)
PDA closed at the time of captopril n (%)	23 (89)
Previous administration of sildenafil n (%)	9 (35)
Gestational age at sildenafil (weeks) (n = 9) ^$^	35 (30, 43.5)
Maximum dose of sildenafil (mg/kg/day) (n = 9)	7 ± 1
Current use of sildenafil n (%)	5 (19)
Previous administration of diuretics n (%)	13 (50)
Current use of diuretics n (%) (hydrochlorothiazide and spironolactone)	8 (31)
Gestational age at first postnatal steroid ^#^ (weeks) (n = 12)	32 ± 4
Duration between steroids and captopril (weeks) (n = 12) ^$^	8 (4, 13)
Postnatal age at captopril administration (days)	105 ± 48
Gestational age at captopril administration (median, range weeks)	40 (37–67)
Gestational age at reassessment (median, range weeks)	45 (42–73)
Ventilator status at discharge (n = 24) *	^ Combined HF-LF = 6
	LF oxygen = 8
	Self-ventilating in room air = 10

* 2 infants died, ^ typically 18 h high flow (HF)–6 h low flow (LF), ^#^ 10-day course of dexamethasone (DART regimen), and ^$^ median (interquartile range).

**Table 2:** Evolution of echocardiographic variables before and five weeks after captopril treatment (n = 26).

Variable	Assessment 1	Assessment 2	*p*
Heart rate (beats/min)	141 ± 7	143 ± 6	0.3
Systolic function			
Fractional shortening (%)	30.4 ± 3.6	33 ± 3	0.01
Mean velocity of circumferential fibre shortening (circ/s)	1.6 ± 0.2	1.9 ± 0.2	<0.0001
Left ventricular output (mL/kg/min)	177 ± 34	230 ± 54	0.0001
Right ventricular output (mL/kg/min)	200 ± 37	232 ± 77	0.07
TDI myocardial performance index (left ventricular)	0.33 ± 0.01	0.29 ± 0.02	<0.0001
Tricuspid annular plane systolic excursion (mm)	8.9 ± 0.7	8.9 ± 0.6	0.0001
Pulmonary vascular resistance index 1/(TPV/RVET)	3.8 ± 0.2	3.5 ± 0.2	0.0002
Diastolic function			
Trans-mitral E/A ratio	1. ± 0.07	0.94 ± 0.07	0.0004
Iso-volumic relaxation time (ms)	65 ± 3.4	56 ± 4.1	<0.0001
Mitral valve stroke volume (mL/kg)	4.8 ± 0.5	5.3 ± 0.6	0.0007
Left atria:aorta ratio	1.7 ± 0.1	1.4 ± 0.1	<0.0001
Pulmonary vein velocity time integral (cm)	8.5 ± 1	10.4 ± 1.2	<0.0001
Vascular parameters			
Aortic intima-media thickness (µm)	877 ± 51	821 ± 71	0.002
Pulsatile diameter (µm)	40 ± 13	50 ± 11	0.005

TDI—tissue Doppler imaging; TPV/RVET—time to peak velocity/right-ventricular ejection time.

## Data Availability

The data presented in this study are available on request from the corresponding author.

## Supplementary Materials

The following supporting information can be downloaded at https://www.mdpi.com/article/10.3390/children13020275/s1, Figure S1: Proposed bronchopulmonary dysplasia pathophysiologic and therapeutic model; Figure S2: Baseline echocardiographic information and its evolution with captopril therapy in one representative infant. Table S1: Pulmonary Hypertension Phenotypes; Table S2: Towards diagnostic and therapeutic precision in BPD.
