# Supplementary material for: Tailoring Therapy to Bronchopulmonary Dysplasia Phenotype: A Ten-Year Experience in Precision Medicine
Source: Children (Basel). 2026 Feb 17;13(2):275. doi: 10.3390/children13020275 (PMC12939007; doi:10.3390/children13020275)
Supplement: Supplementary file 1 [file children-13-00275-s001.zip › Supplementary Table S1.pdf]

Supplementary Table S1. Pulmonary Hypertension Phenotypes.

1. Persistent Pulmonary Hypertension of the Newborn		
Clinical features	Echocardiographic parameters	Management principles
• Oxygenation failure/labile oxygenation<br>• Pre and post-ductal saturation gradient<br>• High oxygenation index<br>• Commonly secondary to meconium aspiration (term) or sepsis/severe HMD (preterm)	• PDA/ASD bidirectional shunt (>30% of cardiac cycle)<br>• ↑1/(TPV/RVET)<br>• ↑LVsEI (septal flattening/bowing)<br>• RV dysfunction<br>• PA PWD notching<br>• Tricuspid regurgitation	• Optimize mean airway pressure (HFO if indicated)<br>• Sedation/muscle relaxation<br>• Selective pulmonary vasodilators (iNO/sildenafil/milrinone)<br>• Prostaglandin E2 for ductal patency
2. High flow systemic to pulmonary shunting (hsPDA or large VSD/ASD)		
• ↑ Pulmonary blood flow<br>• Dilated ventricles (↑ output)<br>• Mostly in premature infants born ≤30 weeks	• Dilated RV if large left to right atrial shunt<br>• Dilated LV & ↑LVO if large hsPDA<br>• May have absent/reversed diastolic Doppler in systemic arteries (ductal steal)	• Diuretics<br>• ↑distending pressure (CPAP/PEEP)<br>• Permissive hypercapnia →↑PVR<br>• Avoid selective pulmonary vasodilators
3. Post-capillary/LHD phenotype (excluding pulmonary vein stenosis and congenital heart disease)		
• Systemic hypertension & systemic artery stiffness	• Dilated/dysfunctional LV<br>• ↑End-diastolic left-atrial pressure	• Trial of diuretics<br>• ↑distending pressure (CPAP/PEEP)

• ↑Systemic afterload<br>• Pulmonary venous congestion	• Left to right shunt if PFO still patent<br>• ↑LV myocardial performance index, ↓mVCF, ↓ PV VTI, ↓MVSV, ↑IVRT, ↓Aortic pulsatility	• Systemic afterload reduction (ACE inhibition)<br>• Avoid pulmonary vasodilators

LV-left ventricle, mVCF-mean velocity of circumferential fibre shortening, PV VTI-pulmonary vein velocity time integral, MVSV-mitral valve stroke volume, IVRT-iso-volumic relaxation time, ASD-atrial septal defect, PFO-patent foramen ovale, CPAP-continuous positive airway pressure, PEEP-positive end-expiratory pressure, RVO-right ventricular output, LVO-left ventricular output, PA-pulmonary artery, PWD-pulse wave Doppler, sEI-systolic eccentricity index, TPV-rime to peak velocity, RVET-right ventricular ejection time, PVR-pulmonary vascular resistance, HFO-high frequency oscillation, hsPDA- haemodynamically significant patent ductus arteriosus, ACE-angiotensin converting enzyme, HMD-hyaline membrane disease.
