# Supplementary material for: Tailoring Therapy to Bronchopulmonary Dysplasia Phenotype: A Ten-Year Experience in Precision Medicine
Source: Children (Basel). 2026 Feb 17;13(2):275. doi: 10.3390/children13020275 (PMC12939007; doi:10.3390/children13020275)
Supplement: Supplementary file 1 [file children-13-00275-s001.zip › Supplementary Table S2.pdf]

Supplementary Table S2: Towards diagnostic and therapeutic precision in BPD

| |
|---|
| a. Attention to LHD-systemic circulation-BPD axis with relevance to pathophysiology and therapeutics. Systemic hypertension is the current investigation trigger.<br>b. Integrated effort from: neonatologists with haemodynamics and lung physiology expertise, respiratory consultants, paediatric cardiologists.<br>c. Attention to echocardiography indices of LHD (**Figure 1**). A dilated LV on 4-chamber apical view might be the first clue of 'post-capillary pathophysiology' (against the expected RV dilatation in conventional pulmonary hypertension).<br>d. Evaluation of aortic pulsatility and indices of stiffness.<br>e. Assessment of reactivity of the pulmonary circulation *before* administration of selective pulmonary vasodilators. Performed using cardiac catheterization or echocardiography, this testing uses iNO and 100% oxygen for a short period.<br>f. Conventional pulmonary vasodilators (such as iNO or sildenafil) that act on the pulmonary arterial circulation are not suited for ongoing management. Essentially, in any condition that restricts blood flow efflux out of the lungs (functional: systemic arterial stiffness/LHD or anatomical: pulmonary vein stenosis/mitral valve disease), they may be counterproductive.<br>g. Better characterization of distinct phenotypes and patient selection may also improve the quality of future randomized clinical trials. |

LHD-left heart dysfunction, LV-left ventricle, iNO-inhaled nitric oxide, RV-right ventricle, BPD-bronchopulmonary dysplasia. References [3, 4, 28].
