# Supplementary figures and images for: Tailoring Therapy to Bronchopulmonary Dysplasia Phenotype: A Ten-Year Experience in Precision Medicine
Source: Children (Basel). 2026 Feb 17;13(2):275. doi: 10.3390/children13020275 (PMC12939007; doi:10.3390/children13020275)

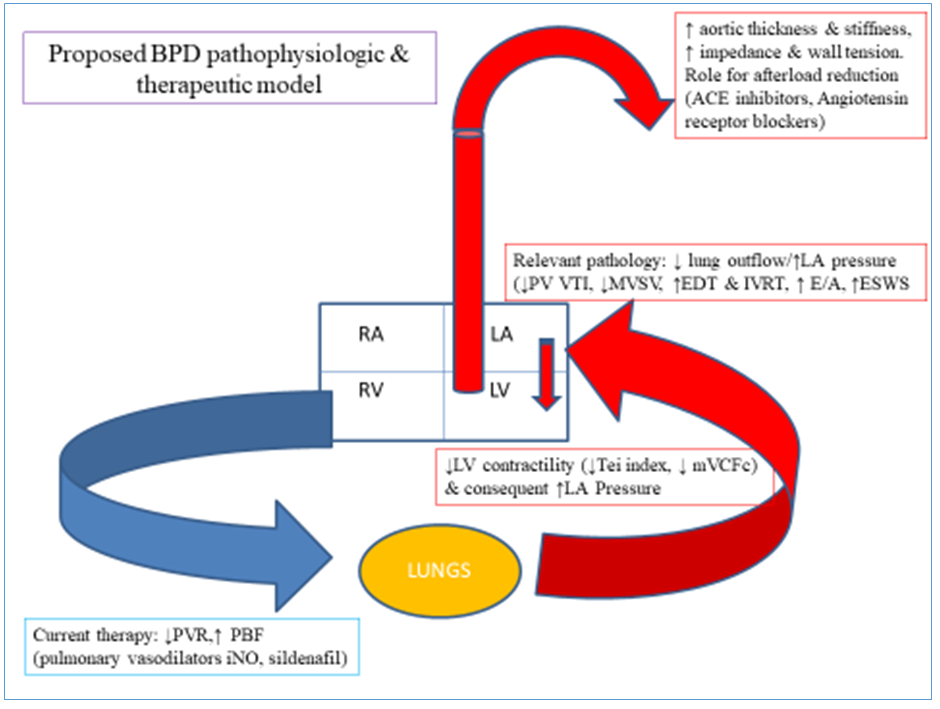

Supplement: Supplementary file 1 [file children-13-00275-s001.zip › Supplementary Figure S1.tif]

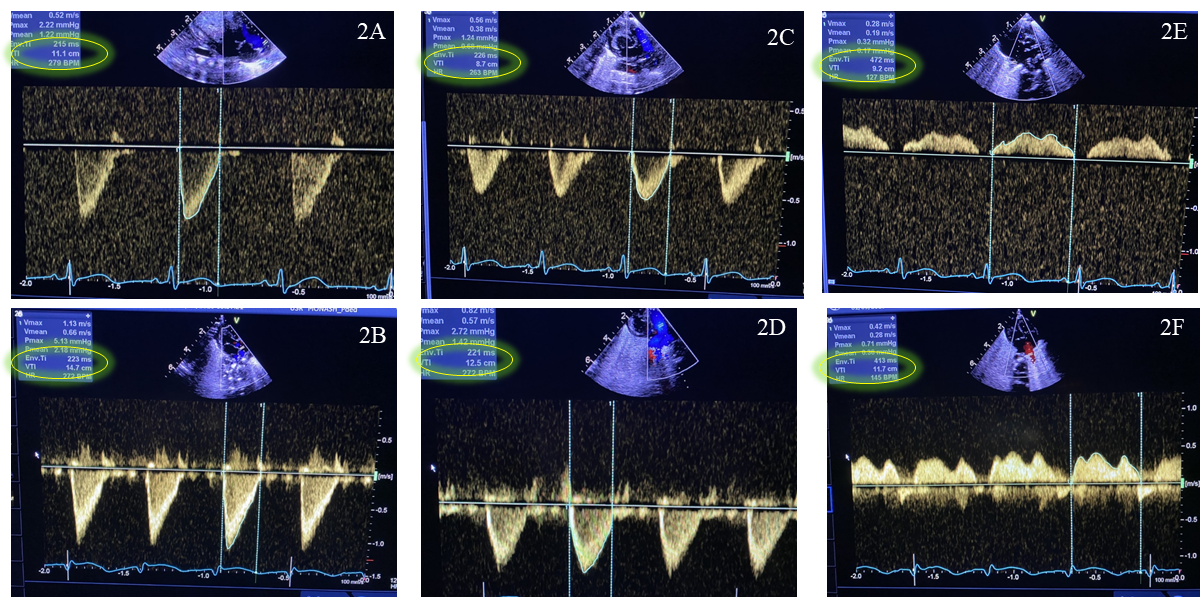

Supplement: Supplementary file 1 [file children-13-00275-s001.zip › Supplementary Figure S2.tif]
